# Psychological workplace violence and its influence on professional commitment among nursing interns in China: A multicenter cross-sectional study

**DOI:** 10.3389/fpubh.2023.1148105

**Published:** 2023-02-27

**Authors:** Zixu Yu, Dong Kong, Yaqin Li, Jie Zhang, Aiwen Guo, Qi Xie, Feng Gao, Xiaoli Luan, Xin Zhuang, Chunling Du, Jin Liu

**Affiliations:** ^1^Department of Nursing, Shandong Provincial Hospital Affiliated to Shandong First Medical University, Jinan, China; ^2^Nursing Department, Shandong Medical College, Jinan, China; ^3^Department of Education and Science, Third People's Hospital, Jinan, China; ^4^School of Nursing, Shandong University of Traditional Chinese Medicine, Jinan, China; ^5^School of Medicine and Nursing, Dezhou University, Dezhou, China; ^6^Department of Neonatology, Shandong Provincial Hospital Affiliated to Shandong First Medical University, Jinan, China

**Keywords:** psychological, workplace violence, professional commitment, nursing interns, China

## Abstract

**Background:**

Psychological workplace violence (WPV) is the primary form of workplace violence suffered by nursing interns. Psychological WPV not only damages the physical and mental health of nursing interns, but also has a negative impact on their work quality and career choice.

**Aim:**

To investigate the characteristics and types of psychological WPV suffered by nursing interns in China, analyze the influencing factors of psychological WPV among nursing interns, and explore the influence of psychological WPV on the professional commitment of nursing interns.

**Methods:**

The subjects were 1,095 nursing interns from 14 medical colleges in Shandong Province. The data were collected electronically using the psychological WPV against nursing interns questionnaire and the professional commitment scale of nursing. The frequency and component ratio were used to describe the incidence and characteristics of psychological WPV. Binary logistic regression was used to analyze the influencing factors of psychological WPV, and linear regression investigated the influence of psychological WPV on the professional commitment of nursing interns.

**Results:**

In the study, 45.0% (*n* = 493) of nursing interns suffered at least one incidence of psychological WPV during clinical practice, mainly discrimination and verbal abuse. Patients and their relatives were the main perpetrators of psychological WPV. Discrimination and lack of trust were the two main reasons behind psychological WPV. Furthermore, 75.9% of psychological WPV incidents were not effectively reported. Logistic regression showed that clinical internship duration, place of family residence, and hospital level were the influencing factors of psychological WPV among nursing interns. Linear regression results showed that psychological WPV had a negative effect on nursing interns' professional commitment.

**Conclusion:**

Psychological WPV against nursing interns is highly prevalent in China, negatively impacting their professional commitment. It is suggested that colleges should introduce courses for nursing interns to understand and cope with psychological WPV before entering clinical practice, and hospitals should establish a mechanism to prevent, cope with, report, and deal with psychological WPV to effectively reduce the incidence of psychological WPV against nursing interns, improve their ability to cope with psychological WPV, and enhance their professional commitment.

## 1. Introduction

The lack of clinical knowledge, nursing skills (1) and ability to deal with clinical emergencies make nursing interns a high-risk group for workplace violence (WPV) (2). WPV is further categorized into physical WPV and psychological WPV. Psychological WPV refers to the deliberate use of power over another person or group, including the use of threats and force, which may cause harm to physical, mental, spiritual, moral, or social development, and includes abuse, bullying/siege, harassment, and threats (3). A UK study of WPV among nursing interns found that about 42.2% of interns had experienced bullying/harassment in the past year (4). A survey on WPV among 1,017 nursing interns in Hong Kong showed that 30.6% had experienced verbal abuse (5). In Turkey, nearly 91.6% of nursing interns had experienced verbal violence (6). In a study of WPV among 954 nursing interns in China, 38.5% had experienced verbal abuse and 14.8% received threats (2). These findings show that psychological WPV has become the primary form of WPV among nursing interns. Researchers believe that the prevalence of psychological WPV is underestimated compared to physical WPV (7).

WPV has many negative physical and psychological consequences for nursing interns (8). A Scottish survey on verbal violence among 950 nursing interns found that anxiety, fear, and vulnerability were the most common symptoms during and after the violence, while a small number of nursing interns felt guilty and incompetent (9). Furthermore, nursing interns exposed to WPV showed higher symptoms of traumatic stress, and their daily life was also affected (10). WPV also has a negative impact on the quality of work and career choices of nurses. A qualitative study by Smith et al. showed that nursing interns who experienced harassment felt that the quality of care provided to patients had decreased (11). Nursing interns' high violence level and experience negatively impact their professional identity, enthusiasm for clinical internship, and work quality (2, 12). Compared with nursing interns who had not experienced verbal violence, those who suffered from verbal abuse had an increased intention to leave (5). About 20% of nursing interns who experienced harassment had considered leaving the nursing profession (13). So far, the current situation and consequences of psychological WPV among nursing interns have not been systematically studied (14).

The professional commitment of nurses refers to the positive attitude and behavior of nurses who identify with their major and are willing to make corresponding efforts, which reflects the status of nurses' identification, loyalty, and devotion to the nursing profession (15, 16). The higher the professional commitment of nurses, the higher the job satisfaction (17, 18), and the lower the work pressure (19) and turnover intention (20, 21). Studies have confirmed that the level of professional commitment of nurses is affected by psychological WPV (22, 23). Nurses exposed to verbal violence had lower levels of professional commitment than nurses who did not suffer verbal violence (24). Furthermore, research suggests that bullying experienced by young nurses influences professional commitment through the mediating role of emotional exhaustion (25). The level of professional commitment of nursing interns can predict the level of professional commitment after they become registered nurses (26). Therefore, it is important to study the professional commitment of nursing interns for the stability of the nursing team and the improvement of the quality of nursing services (27). Previous studies have confirmed that the professional commitment of nursing interns is affected by the clinical environment (28). However, there is little research on whether the professional commitment level of nursing interns is affected by psychological WPV.

This study aims to comprehensively understand the types and characteristics, causes, and coping methods of psychological WPV suffered by nursing interns in China, analyze the influencing factors of psychological WPV among nursing interns, explore the influence of psychological WPV on nursing interns' professional commitment, provide a reference for the development of prevention and treatment measures for psychological WPV against nursing interns, and further improve the level of professional commitment of nursing interns.

## 2. Materials and methods

### 2.1. Participants and data collection

The study is a multi-center cross-sectional study. Medical colleges with a separate nursing department were set as the standard, and 14 medical colleges in Shandong Province were selected. A convenient sampling method was adopted, and junior/senior nursing students who were clinical interns from April 2021 to July 2021 were selected for the survey. Inclusion criteria: (a) the major was nursing; (b) students were clinical interns; (c) clinical practice duration ≥6 months; (d) informed consent. Exclusion criteria: nursing interns who were unable to participate in the survey for various reasons, such as sick leave or personal leave.

The survey was conducted anonymously through the questionnaire star platform (Sojump). The investigators were teachers from the 14 medical colleges and were trained by the researchers before the survey. The investigators explained the purpose and significance of the study and the method of filling in the questionnaire to the respondents, and gave unified guidance for any problems faced by the nursing interns during the survey.

### 2.2. Measures

#### 2.2.1. Demographic factors

Demographic factors included age, sex, educational background, hospital level, duration of clinical internship, whether the nursing intern is an only child, and place of family residence.

#### 2.2.2. Psychological workplace violence questionnaire

By referring to the Chinese Version of Workplace Violence against Nurses Questionnaire compiled by Chen (29), the questionnaire on WPV developed by WHO (30), and the Chinese Version of Workplace Psychologically Violent Behaviors Instrument (31), the researchers compiled the questionnaire on psychological WPV against nursing interns. The questionnaire consists of 12 items divided into two parts. Part one contained types and frequency of psychological WPV suffered by nursing interns (3 items). Part two contained characteristics, coping mechanisms, and effects of psychological WPV suffered by nursing interns (9 items). To ensure the validity of the questionnaire, five experts with experience in WPV were invited to evaluate the content validity. The item-level content validity index was 0.78–0.86, the scale-level content validity index was 0.82, and the Cronbach's alpha coefficient was 0.93.

#### 2.2.3. Professional commitment scale

The professional commitment of nurses scale compiled by Taiwan scholar Lu (15) and later revised was adopted (32). The scale is divided into three dimensions: willingness to make effort (13 items), maintaining as a membership (8 items), and belief in goals and values (5 items), with a total of 26 items. The scale measured an individual's willingness to identify with the nursing profession, devote themselves to the nursing profession, and stay in the nursing profession. The 4-point scale was rated from 1 being “very unsure” to 4 being “very sure.” The total score of professional commitment was the sum of individual item scores. The higher the scale score indicates that nurses have more satisfactory professional commitment. The Pearson correlation coefficient of the retest reliability of the scale after 3 weeks was 0.89, and the Cronbach's alpha coefficient was 0.94. In this study, the Cronbach's alpha coefficient ranged from 0.89–0.95 for the scale and its three dimensions.

### 2.3. Statistical analysis

SPSS 26.0 software was used to analyze the data. Qualitative data were described by frequency and component ratio, while quantitative data were represented by mean ± standard deviation. The Chi-square test and independent samples *t*-test were used to analyze the influence of demographic factors on psychological WPV. The independent samples *t*-test analyzed the differences in professional commitment between the Psychological WPV and no Psychological WPV groups. The influencing factors of psychological WPV were analyzed through binary logistic regression, and the influence of psychological WPV on the professional commitment of nursing interns was analyzed using linear regression. The difference was statistically significant when *p* < 0.05 on both sides.

### 2.4. Ethical considerations

This study was approved by the Ethics Committee of the Provincial Hospital Affiliated to Shandong First Medical University (Ethics Number: 2022-561). All nursing interns in the survey signed an informed consent form. In accordance with the provisions of the Declaration of Helsinki, the personal data of the respondents were kept strictly confidential and their privacy was maintained.

## 3. Results

### 3.1. Participant characteristics

A total of 1,300 questionnaires were sent out and 1,095 valid responses were collected, with an effective response rate of 84.3%. The average age of nursing interns was 21.15 ± 2.50 years and the average internship duration was 7.82 ± 1.65 months. The interns included 951 (86.8%) females. Of the nursing interns, 839 (76.6%) practiced in Grade III-A hospitals; 232 (21.3%) were an only child, and 710 (64.8%) had families living in rural areas. The demographic characteristics of the nursing interns are shown in Table 1.

**Table 1:** Demographic characteristics of nursing interns with and without psychological WPV experience (*N* = 1,095).

**Variable**	**Mean (SD)/number (%)**	**No psychological WPV (*n =* 602)**	**Psychological WPV (*n =* 493)**	**χ^2^/*F***	***P***
Age	21.15 (2.50)	21.20 ± 2.92	21.20 ± 1.85	−0.69	0.49
**Gender**				
Male	144 (13.2)	87 (14.4)	57 (11.5)	1.98	0.16
Female	951 (86.8)	515 (85.5)	436 (88.4)		
Clinical internship duration	7.82 (1.65)	8.04 ± 0.92	7.42 ± 2.17	6.88	<0.01
**Hospital level**				
Grade III-A hospital	839 (76.6)	421 (69.9)	418 (69.4)	33.82	<0.01
Other	256 (23.4)	181 (30.1)	75 (12.4)		
**Educational level**				
<Bachelor degree	557 (50.9)	303 (50.3)	254 (42.2)	0.15	0.7
≥Bachelor degree	538 (49.1)	299 (49.7)	239 (48.5)		
Only child	232 (21.3)	136 (22.6)	96 (19.5)	1.58	0.21
**Family residence**				
Rural	710 (64.8)	368 (61.1)	342 (69.4)	8.08	<0.01
Urban	385 (35.2)	234 (38.9)	151 (30.6)		

WPV, workplace violence.

### 3.2. Types and incidence of psychological WPV against nursing interns

Among the 1,095 nursing interns, 493 (45.0%) had suffered at least one form of psychological WPV during clinical internship. The top three most prevalent forms of psychological WPV included discrimination (618; 56.4%), verbal abuse (387; 35.3%), and being despised and ignored (293; 26.8%); 227 (20.7%) nursing interns suffered more than two types of psychological WPV. The results are shown in Table 2.

**Table 2:** Incidence rate of psychological WPV (*N* = 1,095).

**Variable**	**Number (%)**
**Psychological WPV**	
Yes	493 (45.0)
No	602 (55.0)
**Types of psychological WPV**	
Discrimination	618 (56.4)
Verbal abuse	387 (35.3)
To despise and ignore	293 (26.8)
Bullying	105 (9.6)
Isolation and alienation	73 (6.7)
Harassment	70 (6.4)
other	77 (7.0)
**Frequency**	
0	602 (55.0)
1	266 (24.3)
2–3	144 (13.1)
>3	83 (7.6)

WPV, workplace violence.

### 3.3. Characteristics, coping mechanisms, and influence of psychological WPV suffered by nursing interns

In further analysis of psychological WPV against the 493 nursing interns, it was found that 54.8% of psychological WPV incidents occurred in the first 1–3 months of clinical internship. The top three perpetrators of psychological WPV were relatives of patients (386; 78.3%), patients (374; 75.9%), and nurses (184; 37.3%). Majority of the psychological WPV incidents (419; 85.0%) occurred between 8:00 to 16:59 h. Furthermore, 56.9% cases of psychological WPV could be attributed to discrimination against the nursing interns and 34.4% cases indicated a lack of trust in their abilities. In the face of psychological WPV, 50.9% of nursing interns chose silence, and 75.9% of violent incidents were not reported. The results are shown in Table 3.

**Table 3:** Characteristics of psychological WPV (*n* = 493).

**Variable**	**Number (%)**
**Internship duration**	
1–3 months	270 (54.8)
4–6 months	187 (37.9)
>6 months	98 (19.9)
**Department**	
Emergency department	254 (51.5)
Outpatient department	203 (41.2)
Inpatient ward	155 (31.4)
Intensive care unit	67 (13.5)
Other	58 (11.8)
**Perpetrator**	
Relatives of patient	386 (78.3)
Patient	374 (75.9)
Nurse/head nurse	233 (47.2)
Doctor	81 (16.4)
Cleaner/security	68 (13.8)
Nurse students	35 (7.1)
Other	101 (20.4)
**Occurrence time**	
8:00–16:59	419 (85.0)
17:00–21:59	74 (15.0)
22:00–7:59	63 (12.8)
**Cause**	
Discrimination against nursing interns	280 (56.9)
Lack of trust in nursing interns	170 (34.4)
Nursing interns' lack of effective communication	101 (20.6)
Low-level education of perpetrator	118 (23.9)
Nursing interns' lack of knowledge and skills	97 (19.6)
No reason	54 (11.0)
other	40 (8.1)
**Coping strategies**	
Suffer in silence	251 (50.9)
Ask classmates/friends for help	168 (34.1)
Patiently explained	152 (30.8)
Ask nursing teacher for help	121 (24.5)
Ask family for help	64 (13.0)
Treat the perpetrator in the same way	33 (6.7)
Other	197 (40.0)
**Report situation**	
No report	374 (75.9)
Intern captain/classmates	183 (37.1)
Nursing teacher	140 (28.4)
Head nurse/nursing department	45 (9.1)
College teacher	26 (5.3)
**Reason for not reporting**	
Normal phenomenon	265 (53.8)
Reporting doesn't solve the problem	168 (34.1)
Unwilling to report	120 (24.3)
No reporting mechanism	71 (14.4)
Fear of retaliation	64 (13.0)
Fear of ridicule	17 (3.4)
Other	9 (1.8)
**Impact of WPV**	
Decline in work enthusiasm	356 (72.2)
Want to leave the department	259 (52.5)
Want to change profession	191 (38.7)
No influence	185 (37.5)
Absenteeism	52 (10.5)
Nursing errors and increase in mistakes	42 (8.5)
Other	70 (14.2)

WPV, workplace violence.

### 3.4. Characteristics of nursing interns with and without psychological WPV experience

The univariate analysis showed that the incidence of psychological WPV was higher among nursing interns with short internship duration, who practiced in Grade III-A hospitals, and who lived in rural areas than those with long internship duration, who practiced in hospitals below Grade III-A hospitals, and who lived in cities (*p* < 0.01 for all parameters). The results are shown in Table 1.

### 3.5. Professional commitment of nursing interns with and without psychological WPV experience

The *t*-test results showed that the scores of professional commitment were higher for nursing interns who did not suffer psychological WPV than for those who experienced psychological WPV (*p* < 0.01). The results are shown in Table 4.

**Table 4:** Professional commitment of nursing interns with and without psychological WPV experience.

**Variable**	**Average score**	**No-WPV (*n =* 602)**	**WPV (*n =* 493)**	***F***	***P***
Total scale score	2.65 ± 0.69	2.72 ± 0.67	2.57 ± 0.73	3.41	0.00
Willingness to make effort	2.71 ± 0.80	2.79 ± 0.77	2.62 ± 0.82	3.48	0.00
Maintaining as a membership	2.61 ± 0.82	2.69 ± 0.78	2.51 ± 0.86	3.58	0.00
Belief in goals and values	2.91 ± 0.77	2.97 ± 0.74	2.83 ± 0.8	3.00	0.00

WPV, workplace violence.

### 3.6. Logistic regression results for factors influencing psychological WPV in nursing interns

Logistic regression analysis was conducted to explore the predictive factors of psychological WPV among nursing interns. Psychological WPV was the dependent variable, and internship duration, internship hospital, place of family residence, and participation in activities were taken as independent variables. The results showed that long duration of clinical practice and living in urban areas were the protective factors of psychological WPV among nursing interns, and practice in Grade III-A hospitals was the risk factor for psychological WPV (Table 5).

**Table 5:** Factors influencing psychological WPV among nursing interns (*N* = 1,095).

	***B***	**S.E**.	**Wald**	***P***	**Exp (B)**	**95% CI**	
Grade III-A hospital	0.56	0.16	11.70	<0.01	1.75	1.27	2.41
Clinical internship duration	−0.05	0.06	65.67	<0.01	0.60	0.53	0.68
Place of family residence—Urban	−0.30	0.14	4.74	0.03	0.74	0.56	0.97
Constant	2.79	0.65	18.48	<0.01	16.24		

CI, confidence interval; WPV, workplace violence.

### 3.7. Linear regression results for the impact of psychological WPV on nursing interns' professional commitment

Multivariate linear regression analysis showed that psychological WPV influenced nursing interns' professional commitment and associated dimensions. See Table 6 for details.

**Table 6:** Influence of psychological WPV on professional commitment of nursing interns.

	***B***	**SE**	**Beta**	***T***	***P***
Professional commitment	−0.19	0.04	−0.14	−4.37	<0.01
Willingness to make an effort	−0.23	0.05	−0.14	−4.53	<0.01
Entry into professional career	−0.23	0.05	−0.14	−4.43	<0.01
Professional value identification	−0.19	0.05	−0.12	−3.39	<0.01

WPV, workplace violence.

Adjust for age, sex, only child, educational level, clinical internship duration, place of family residence.

## 4. Discussion

The study investigated 1,095 nursing interns in 14 medical colleges in the Shandong Province of China, which contains 35 hospitals in 20 cities, representing to a certain extent the incidence level of psychological WPV against nursing interns in China. The results showed a psychological WPV incidence rate of 45.0% among Chinese nursing interns, which is similar to the findings for nursing interns in Australia (33) and the United Kingdom (4), and is higher than the incidence (28.1%−31.3%) of psychological WPV against Chinese nurses (31, 34). This result could be attributed to the inconsistent survey tools used in the investigation. Moreover, compared with nursing interns, nurses have richer clinical experience and are more likely to gain the trust of patients and their families when dealing with clinical routine or emergency problems. The higher the trust in nurses' abilities, the lower the incidence of violence (35). As for the type of psychological violence, discrimination ranks first, followed by verbal abuse, which is inconsistent with other research results (9, 36). This is because patients and their families prefer to be cared for by experienced nurses, which affects the clinical operation opportunities of nursing interns and makes them feel discriminated against. This may also be why nursing interns chose patients and their families as the main perpetrators of psychological WPV. Notably, nurses accounted for more than a third of the perpetrators, indicating that horizontal violence within organizations is widespread amongst nursing interns (37, 38). The first 3 months of the internship are the high-incidence period of psychological WPV, which is the stage of clinical learning and adaptation for nursing interns, preventing nursing interns from comprehensively imbibing the skills required to deal with clinical problems. Most of the psychological WPV incidents against nursing interns occurred during 8:00–16:59 h, which could be attributed to the heavy nursing workload and relative shortage of nurses in China (39). When patients' needs are not met, the incidence of violence significantly increases (40).

When faced with psychological WPV, most of the nursing interns chose silence and tolerance; only 24.1% of the nursing interns reported psychological WPV. The reason why nursing interns choose not to report Psychological WPV is the belief that it is a common phenomenon in clinical practice and will not be dealt with justly after reporting. These findings suggest that the current clinical environment of nursing interns needs to be improved as there is no comprehensive mechanism for preventing, reporting, and dealing with psychological WPV for nursing interns. Moreover, the nursing interns were punished by the leaders/teachers after reporting incidents of psychological WPV (9), resulting in the failure to take appropriate measures to deal with psychological WPV. Faced with psychological WPV, nearly three-quarters of nursing interns' enthusiasm for work decreased, more than half hoped to change the department, and one-third had the idea of changing careers. This indicates that although psychological WPV does not directly damage the health of nursing interns (41), it greatly reduces the confidence and enthusiasm of nursing interns for nursing work and negatively affects their professional identity. This was confirmed through the analysis of influencing factors of professional commitment in this study.

The regression analysis of psychological WPV showed that the duration of clinical internship and family residence in urban areas were protective factors of psychological WPV. In the long-term clinical practice, nursing interns can master more clinical skills and correctly deal with patients' problems, reducing the risk of psychological WPV. In China, compared with nursing interns living in rural areas, interns living in cities have better educational resources (42), enabling them to develop more comprehensive coping skills and strain capacity. These skills have certain advantages in dealing with nurse-patient conflicts; thus, these nursing interns are less likely to suffer from psychological WPV. Compared with nursing interns in lower-level hospitals, nursing interns in Grade III-A hospitals are more likely to suffer psychological WPV. This could be attributable to the fact that patients have more demands and higher treatment expectations from the medical staff in higher-level hospitals, and nursing interns who lack comprehensive knowledge and skills find it difficult to meet patient needs. This is consistent with the finding that lower patient satisfaction leads to higher psychological WPV (43).

This study showed that the professional commitment of nursing interns was at a medium level, which was consistent with other research results (23, 44). The group with experience of psychological WPV had lower overall scores in professional commitment and its three dimensions than the group with no experience of psychological WPV. The regression results also showed that psychological WPV affects the professional commitment of nursing interns. The higher the level of psychological WPV, the lower the level of professional commitment, which is consistent with the results of studies on nurses (22, 23). According to Ecological Systems Theory, individual development is caused by the interaction between oneself and the environment (45). The professional commitment of nursing interns is bound to be affected by the clinical internship environment. Psychological WPV, as an adverse factor, will affect the relationship between nursing interns and patients. The worse the nurse-patient relationship, the lower the professional commitment level (46).

This study find that lack of trust is one of the main reasons for the high incidence of psychological WPV among nursing interns, and effective nurse-patient communication can improve the nurse-patient relationship and reduce the incidence of psychological WPV. Compared with other nurse-patient communication training, researchers found that the use of phenomenologically-based communication training for nursing interns has more advantages (47), which can effectively improve the trust relationship between nursing interns and patients. Effective post-incident support can improve employee outcomes after WPV occurs (48), a greater emphasis on supportive organizational practices are required for reduce the outcomes. It is suggested that hospital administrators formulate perfect procedures for the prevention, reporting, and handling of psychological WPV so as to create a good learning environment for nursing interns. Nursing interns who suffer from psychological WPV should be provided with regular psychological counseling to ensure their physical and mental health (24) so as to enhance their professional commitment to nursing.

The study also has some limitations. First, this study is a cross-sectional study using a convenient sampling method. Information is not available on nursing interns who decided not to participate in the study, so our study may have selective bias. Meanwhile, the nursing interns may have reported only those incidents of psychological WPV which hurt them deeply; therefore, the actual incidence rate of psychological WPV may be higher than that reported in this study. Future studies should conduct prospective data collection on psychological WPV among nursing interns. Secondly, the influence of psychological WPV on the career of nursing interns is multifaceted. This study only discusses the influence on professional commitment; whether psychological WPV affects the employment choice of nursing interns is the subject of future research.

## 5. Conclusion

About half of Chinese nursing interns experience psychological WPV, with discrimination and verbal abuse being the primary forms of psychological WPV. Furthermore, the majority of psychological WPV incidents are not reported. The level of psychological WPV suffered by nursing interns differs based on hospital level, clinical practice duration, and place of family residence. Psychological WPV not only reduces the work enthusiasm of nursing interns, but also affects their professional commitment to nursing. It is suggested that colleges should add courses on understanding and coping with psychological WPV for nursing interns, and hospitals should encourage nursing interns to report violent incidents so as to build a healthy and harmonious professional environment. Meanwhile, managers should take targeted measures to prevent and deal with violence to stabilize nursing teams.

## Data availability statement

The raw data supporting the conclusions of this article will be made available by the authors, without undue reservation.

## Ethics statement

This study was approved by the Ethics Committee of the Provincial Hospital Affiliated to Shandong First Medical University (Ethics Number: 2022-561). All nursing interns in the survey signed an informed consent form. In accordance with the provisions of the Declaration of Helsinki, the personal data of the respondents were kept strictly confidential and their privacy was maintained.

## Author contributions

JL and DK contributed to the study design, implementation, and analysis. JL, YL, and ZY contributed to manuscript writing. JZ, QX, FG, and AG contributed to the statistical design and data collection. XL, XZ, and CD contributed to the analysis of the data. All authors contributed to the article and approved the submitted version.
